# Neuromodulation in 2035: The Neurology Future Forecasting Series

**DOI:** 10.1212/WNL.0000000000013061

**Published:** 2022-01-11

**Authors:** Tim Denison, Martha J. Morrell

**Affiliations:** From the Department of Engineering Science (T.D.), University of Oxford, UK; Department of Neurology and Neurological Sciences (M.J.M), Stanford University, CA; and NeuroPace (M.J.M), Mountain View, CA.

## Abstract

Neuromodulation devices are approved in the United States for the treatment of movement disorders, epilepsy, pain, and depression, and are used off-label for other neurologic indications. By 2035, advances in our understanding of neuroanatomical networks and in the mechanism of action of stimulation, coupled with developments in material science, miniaturization, energy storage, and delivery, will expand the use of neuromodulation devices. Neuromodulation approaches are flexible and modifiable. Stimulation can be targeted to a dysfunctional brain focus, region, or network, and can be delivered as a single treatment, continuously, according to a duty cycle, or in response to physiologic changes. Programming can be titrated and modified based on the clinical response or a physiologic biomarker. In addition to keeping pace with clinical and technological developments, neurologists in 2035 will need to navigate complex ethical and economic considerations to ensure access to neuromodulation technology for a rapidly expanding population of patients. This article provides an overview of systems in use today and those that are anticipated and highlights the opportunities and challenges for the future, some of which are technical, but most of which will be addressed by learning about brain networks, and from rapidly growing experience with neuromodulation devices.

The treatment of many disorders of the nervous system relies on chronic noncurative pharmacologic or biologic treatments or on creation of focal lesions. Neuromodulation therapies offer another approach: targeting and disrupting a dysfunctional brain focus, region, or network. For purposes of brevity, we use the term neuromodulation broadly, to include direct stimulation of a neural substrate to drive action potentials and modulate distributed neural activity, as well as subthreshold stimulation to bias local activity.

## Clinical Applications

The history of using “bioelectricity” to treat disorders of the nervous system dates to at least 2750 BC, starting with electric fish, evolving to suitcase-sized cardiac pacemakers, and then to implanted spinal cord and brain stimulation devices. “Brain pacemakers” emerged in the 1980s to treat the symptoms of Parkinson disease^1^ and essential tremor^2^ by providing stimulation to alter pathologic neuronal circuits. Deep brain stimulation (DBS) was subsequently found effective for dystonia,^3^ obsessive-compulsive disorder (OCD),^4^ and focal epilepsy.^5^ Direct stimulation of the ascending vagus nerve was approved by the US Food and Drug Administration (FDA) for medically intractable focal epilepsy in 1997 and later for depression.^6,7^ Transcranial magnetic stimulation was approved for the treatment of major depression in 2008 and for treating pain with certain migraine headaches in 2013.^8^ Advances in technology in the 2000s brought the first responsive neurostimulator for treating epilepsy, which uses embedded amplifiers and algorithms to sense and detect abnormal bioelectrical waveforms off implanted electrodes, and then apply targeted brain stimulation in response.^9^

**Figure 1:**
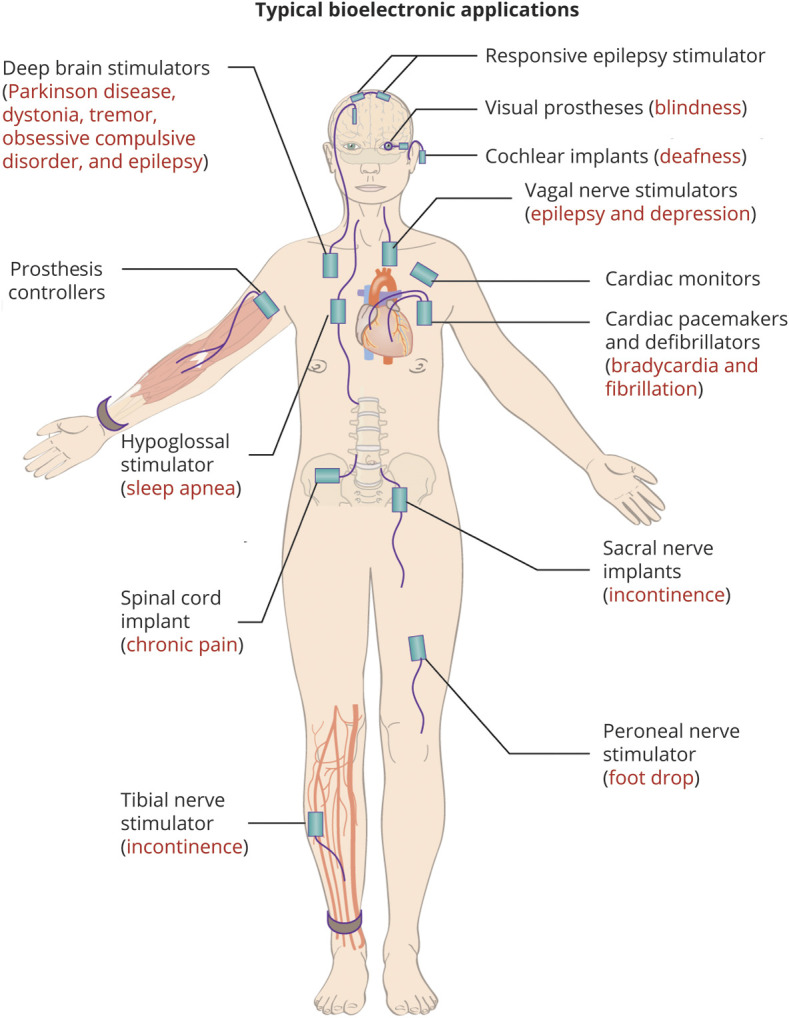
Examples of Clinical Applications Using Electrical Stimulation Devices The device location is dictated by the anatomy of the “target.” Note that the same area of the nervous system can be a common target for multiple disorders, such as the basal ganglia for Parkinson disease and dystonia. Other disorders may have multiple targets, such as epilepsy.

As illustrated in Figure 1, external and implantable neuromodulation devices are available to treat a number of symptoms and disorders at multiple targets and levels of the nervous system. A partial list of neuromodulation devices that are FDA-approved or investigational for treatment of symptoms and disorders of the nervous system is provided in the Table.

**Table:**
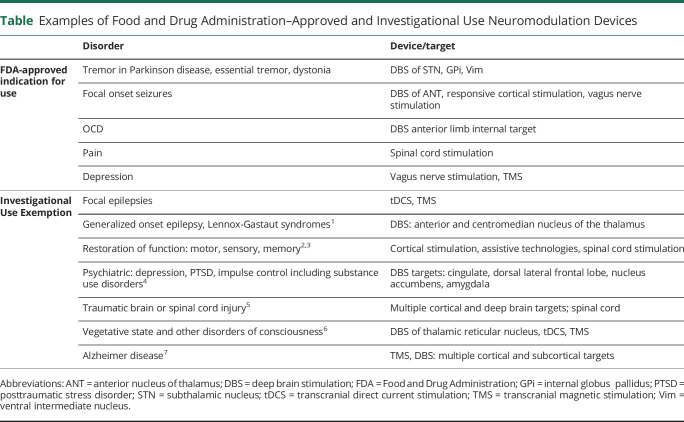
Examples of Food and Drug Administration–Approved and Investigational Use Neuromodulation Devices

There is enormous opportunity to improve technology and explore new approaches and potential applications, including for poststroke motor rehabilitation,^10^ memory disorders,^11^ mood disorders,^12^ brain and spinal cord trauma,^13^ prolonged disorders of consciousness,^14^ and Alzheimer disease.^15^ Neuroscience discovery into mechanisms of action of different approaches, combined with advancements in areas such as computational capabilities, miniaturization, and materials science, are enabling development of new device therapies.^16,17^ Development will accelerate as clinicians, recognizing the clinical need, integrate devices into routine patient care.

## Foundations: Modulating and Stimulating the Nervous System

The method for modulating the nervous system is often a trade-off among specificity of location, invasiveness, and patient acceptability. Noninvasive methods have the advantage of requiring no surgery, but may require a bulky wearable appendage or repeated clinic visits; an implant is generally able to provide more spatial specificity and require less patient interaction. As with most engineered systems, neuromodulation devices often have conflicting constraints, and systems are designed to balance these trade-offs and provide a variety of options for the patient and clinician.

Current noninvasive systems rely on electrical activation of the nervous system without compromising the skin. Transcranial direct current stimulation (tDCS) drives a DC current between at least 2 external electrodes. The proposed mechanism is that neural activity is promoted by the current flow under one electrode, and is inhibited under the second^18^; clinical applications are being explored in rehabilitation, neuropsychiatry, epilepsy, and memory enhancement.^19,20^ A derivative of this technique is transcranial alternating current stimulation (tACS). tACS provides a variable excitation waveform that can mimic natural brain rhythms such as the theta band (∼4 Hz) associated with memory, create high-frequency impulses for blocking pain, or explore the effect on dynamic motor systems.^21^ Similar to tDCS, the choice of electrode placement is important to engage the desired neural circuits.

A challenge with both tACS and tDCS is getting currents to penetrate to the targeted neural circuit without causing skin irritation. One method for addressing this is to use transcranial magnetic stimulation (TMS). TMS uses a high current pulse through a coil placed over a specified region of the skin. The current pulses create a large magnetic field (approximately 1 T for peak over 100 μS), which induces a countercurrent that can excite the nervous system. TMS is used investigationally for mapping the brain before surgery and is approved for treatment of depression and some forms of migraine.^8^ In the future, more advanced transcranial techniques might be applied. One example is temporal interference, which superimposes different stimulation patterns to deliver therapy more deeply in the brain.^22^ Another is “paired stimulation,” which aims to synchronize different regions of the brain and leverage the concept that “neurons that fire together wire together.”^23^

For greater specificity, invasive stimulation places the electrode in the vicinity of the neural substrate. The critical consideration for invasive stimulation is the tissue–electrode interface. When an electrode is placed inside a physiologic medium such as neural tissue, an interface forms between the 2 media and charge carries from electrons in the electrode to ions (such as Na^+^, K^+^, Cl^−^) in the tissue, either by a capacitive mechanism (nonfaradaic reaction) or by reduction or oxidation reactions (faradaic reaction).^24^ The charge transferred depends on stimulation paradigms, material properties, tissue characteristics, and other variables. For implants, nonfaradaic reactions are generally preferred to avoid electrode or tissue damage. When the appropriate materials and stimulation measures are chosen, the electrode–tissue interface remains stable, enabling reliability of stimulating electrodes over the long term.^25^

## Emerging Technical and Therapeutic Opportunities

**Figure 2:**
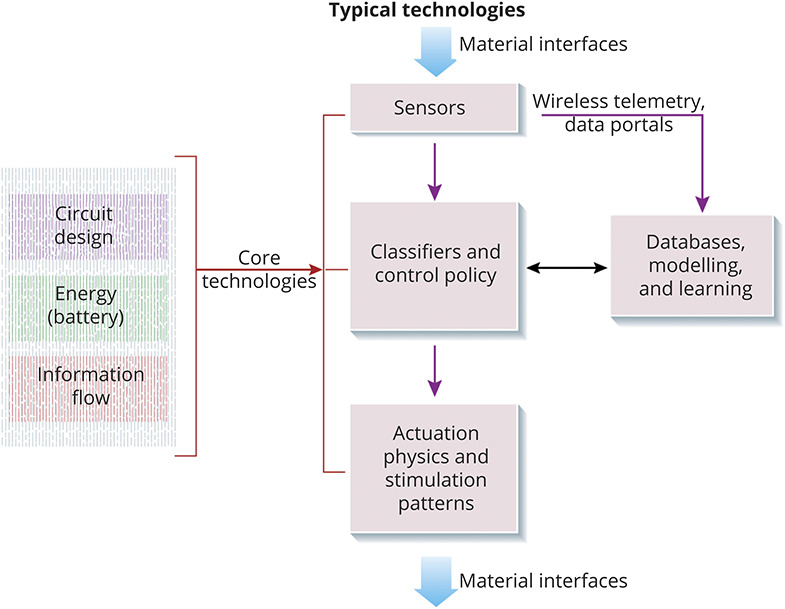
Technology Stack for a Neuromodulation Device The system interacts with the nervous system through material interfaces for sensing physiologic signals as well as stimulation. The sensors can include direct measurements of electrical activity or surrogates such as inertial signals. These signals can be used to estimate the patient's state, and then a control policy determines what adjustments should be made to the stimulation pattern. The closed-loop system is supported with databases, modeling, and machine learning to optimize performance; data gathering requires additional infrastructure for telemetry and data storage. Core technologies such as circuit design, energy storage, and information are common to many stack designs.

Multiple technical challenges, ranging from materials science to battery technology to electronics to information security, must be resolved to design a successful medical implant. This is referred to as the technology stack,^26^ and is illustrated in Figure 2. For example, the materials interface must not cause inflammation or harm the surrounding tissue (biocompatibility), and the harsh biological environment—warm and corrosive—must not harm the implant (biostability); a typical implant is expected to last more than a decade. Materials are also critical in design of leads that receive signals for processing and route stimulation to the neural circuits. In addition, the energy requirements of an implanted device, approximately hundreds of microwatts to milliwatts, motivate new microelectronics, battery technology, and miniaturization, which have benefited from advancements in consumer technology.

The majority of implanted neuromodulation devices currently run in an “open-loop” mode of operation, providing a train of impulses to a specific anatomic target continuously or on a fixed duty cycle. Stimulation settings are selected by a clinician. The patient might have some marginal control to adjust stimulation or turn the system on and off.

Responsive, or closed-loop, neuromodulation systems adjust stimulation according to a clinically relevant physiologic signal. Because some seizures are associated with acceleration in heart rate, a heart rate sensor incorporated into some vagus nerve stimulation devices activates stimulation when heart rate exceeds a predetermined threshold.^27^ A spinal cord stimulation system uses an embedded 3-axis accelerometer to dynamically adjust the stimulation amplitude based on changes in posture and activity.^28^ Another example is a responsive neurostimulator for the treatment of epilepsy^9^ that continuously monitors intracranial EEG using electrodes placed in the region of seizure onset. Stimulation is provided only when epileptiform activity is detected, reducing the amount of stimulation from hours a day, as is the case for closed-loop devices, to an average of about 3 minutes per day. Devices can also respond to evoked potentials and adjust stimulation on a pulse-per-pulse basis.^34^ This optimization approach is being explored for the improvement of spinal cord stimulation.

Clinical trials are underway for adaptive DBS in Parkinson disease. The model is to titrate stimulation according to aberrant oscillations in the basal ganglia. When oscillations exceed a threshold amplitude, stimulation is titrated upward. When there is a reduction in oscillations—for example, when medication is taken—stimulation is turned down. Similar concepts are being explored for depression, obsessive-compulsive disorder, and essential tremor.^29^

**Figure 3:**
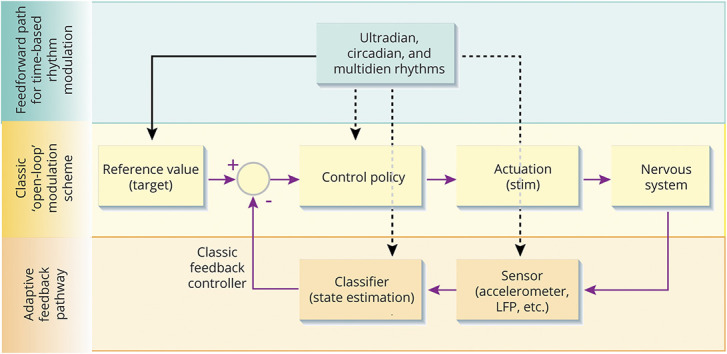
Device Approaches to Optimize Therapeutic Control Future device algorithms will combine multiple approaches to optimize therapeutic control. Similar to physiologic processes, the devices will optimize predictive, feedforward models and responsive, sensor-based feedback algorithms. The middle open loop signal flow represents classical stimulation methods, in which the clinician acts as the control for configuring the stimulator based on immediate observations. Recently, the adaptive feedback methods use embedded sensors to adjust stimulation in real-time, with the know-how of the clinician applied in an algorithm that classifies the patient's state, and then takes the appropriate action with the control policy 24 hours a day, 7 days a week. Researchers are now exploring how feedforward mechanisms such as sleep–wake and other biological rhythms at multiple timescales might optimize control of the device.

Stimulation might also be adapted according to time-based biological rhythms. Research is underway in patients with epilepsy to understand how to modify responsive stimulation according to individual circadian/diurnal and multiday seizure rhythms and to forecast times of greater seizure susceptibility.^30,31^ Similar efforts seek to modify diurnal stimulation for movement disorders based on that patient's sleep–wake cycle. In the future, as mapped in Figure 3, the algorithms in devices will integrate both circadian feedforward adjustments to the stimulation pattern and short-time responsive modes, much like natural control mechanisms work to regulate physiology.^32^

## Forecasting Developments: First, Better Understanding of the Neural Network

The therapeutic power of neuromodulation comes from the ability to target and modulate a specific network. However, even for a given symptom, that network may not be the same from patient to patient, dysfunction within a specific network could cause different symptoms, and there may be multiple networks expressing multiple symptoms within a single syndrome. One example is Parkinson disease, in which stimulation of the subthalamic nucleus treats tremor, while stimulation of the pedunculopontine nucleus might address gait freezing and axial instability. Stimulation of the spinal cord or motor cortex relieves the phenomenon of pain, but stimulation of the prefrontal cortex may be necessary to relieve the distress of pain. Dysfunction of the amygdala may be expressed as seizures, posttraumatic stress disorder (PTSD), or other behavioral disorders. Disruption of networks that include the ventral striatum/nucleus accumbens may be expressed as an impulse control disorder, such as addiction or binge eating; as OCD; or as a mood disorder, such as depression. By understanding the network disruption that leads to symptoms for each patient, personalized, targeted, efficient, and effective interventions can be devised. The premise is that even when patients are phenotype-similar, they may be network-dissimilar. There will be no one-size-fits-all approach.

Key challenges to effective treatment are to define the targets for stimulation and the “dose,” recognizing there are multiple configurable stimulation measures, including stimulation pathways, frequency, pulse width, duration, current, and whether the stimulation is provided as a single treatment, continuously, on a duty cycle or responsively. Another consideration is the treatment duration. For many neuromodulation therapies, clinical improvements are increasingly evident over time measured in months, as in recovery after stroke, or years, as with epilepsy. This suggests that favorable clinical effects are mediated not only by acute responses to stimulation but also by longer-term changes in neuroplasticity. As in chronobiology, a better understanding of the temporal characteristics of neuromodulation is critical to its optimization.

One of the neurologist's roles is to use clinical acumen to localize the dysfunctional network underlying the disorder for each patient. The development of more refined neural network models of neurologic and psychiatric disorders will be aided by advancements in electrophysiology and in structural and functional imaging, such as fMRI and diffusion tensor imaging, and by the development of more precise and less invasive brain mapping techniques, such as TMS, or new stereotactic electrode implantation techniques.

## The Increasing Value of Data

The neurologist is trained to obtain a meticulous history and perform a thorough examination in order to form a differential diagnosis, identify appropriate additional diagnostic testing, and establish a treatment plan. Thereafter, the success of treatment is generally determined by the patient's report of symptoms. However, symptoms may be a lagging indicator of disease progression or remission, and in many cases, patients are not able to reliably report their symptoms. Examples include the patient with epilepsy who has seizures while asleep, or the patient with a dementing illness and poor insight. This leaves the neurologist to make significant treatment decisions based on incomplete and potentially inaccurate data. Contrast this to the endocrinologist, who designs treatment regimens based on continuous glucose monitoring, or the cardiologist, who has continuous heart rate monitoring to inform treatment of paroxysmal atrial fibrillation.

The goal is to provide the neurologist with biomarkers that indicate physiologic changes that precede the clinical consequences of a disease. The neurophysiologic information obtained from today's sensing-enabled devices is fueling discovery of neurologic and psychiatric biomarkers. Biomarkers are already identified for a number of disorders, such as beta frequencies in Parkinson disease and essential tremor,^33^ or spikes in persons with epilepsy.^34^ Preliminary data suggest that there are biomarkers that precede the tic in Tourette syndrome, the urge to binge in persons with loss of control over eating, and before panic episodes in PTSD.

The volume and complexity of the data provided by these devices require methods for interpretation that do not depend on the clinician, no matter how skilled that clinician may be. Interpreting brain data, particularly data obtained chronically or in real time, requires advanced analytics that often rely on machine and deep learning and require intense computational capabilities.^35^ Neurologic therapies, such as neuromodulation, will increasingly rely on data science to achieve best outcomes.

## Optimizing Stimulation Methods

A substantial challenge in bioelectronic design is our relatively poor understanding of how the nervous system operates. To this end, bioelectronic platforms are incorporating instrumentation to chronically access neural information and identify objective biomarkers of pathologic and normal function. Several programs, such as the NIH BRAIN and SPARC initiatives, are actively supporting the research and technology required to exploit this neurophysiologic data to better understand disease mechanisms and to develop new therapies.

Innovations in fundamental interfaces will continue. Future methods of modulation will aim for greater specificity and less invasiveness. Focused ultrasound is being explored as a way to mechanically actuate the nervous system by focusing energy deep in the brain with a noninvasive transducer.^36^ A disadvantage of focused ultrasound is the need for a large external transducer, which would be awkward to use outside a controlled environment.

In the longer term, optogenetics might be an approach for greater specificity in neural actuation.^37^ Neurons are modified to express light-sensitive opsins and then subsequently controlled by light.^37^ The potential therapeutic advantage of optogenetics is the specificity to cell types, the ability to modulate different cell types with different wavelengths, and the capability to directly inhibit or excite activity. Translation to humans faces several obstacles, including concerns about using viral vectors to deliver the opsin, the extreme power requirements required to reach the threshold of optical excitation, the need for optical routing to a target, and concerns for phototoxicity. At present, optogenetics is being used as a tool to understand networks in conditions such as in Parkinson disease and neuropsychiatric disorders, and to guide more traditional methods such as DBS and TMS.

## Future State Considerations

### Data Security and Privacy

Some neuromodulation devices detect, digitize, interpret, and act on information contained in neural activity systems. Systems must be developed to guard against this data being abused or hacked. Issues to be addressed include how long and where these data should be stored and who is in charge of it. If data can be “written to” the brain, we need systems to guard against undesirable intrusions, for example in the form of advertising or political influence.

### Health Care Economics

In order to be available to patients, a device therapy must demonstrate that it addresses a clinical need, is safe, effective, reliable, and easy to use in routine clinical practice, and provides value within the context of health care economics. The hospital, the neurologist and surgeon, as well as the developer and provider of the device must be adequately compensated. Reimbursement policies must keep abreast of rapid developments in technology and data science, and consider not only the costs of the device and procedure, but the time and expertise of the neurologist interpreting complex data sets and managing multifunction devices. Economics can also drive decisions in the technology stack, specifically looking for opportunities to leverage core building blocks from other industries and repurposing proven devices for new therapy opportunities.

### Regulatory Challenges

Devices are regulated by the Center for Devices and Regulatory Health. Compared to pharmacologic studies, device studies tend to have fewer participants with longer follow-up (often years). For novel devices, an initial feasibility study is generally conducted in order to show safety and provide some evidence of efficacy. The majority of devices then require a blinded, randomized controlled study to demonstrate safety and effectiveness. A challenge for some device studies is to maintain a blind, since it is not always possible to mask the perception of the stimulation. Other regulatory requirements are to provide data on how the skill of the physician user affects patient outcomes and to show that the user interface is acceptable to physician and patient. Although device trials tend to be less costly than pharmaceutical trials, multiple millions of dollars are still required to move a new device from concept, technology development, clinical trials and FDA approval, to the patient. As with other therapies, data specific to genetics, sex, children, and underrepresented communities are often lacking.

The rate of development in technology may outpace the rate at which regulatory agencies can assess safety and effectiveness using traditional models of regulatory science. Cellular phones change their operating systems frequently, but medical devices, especially those that are implanted, must ensure that any changes to hardware, firmware, or software do not affect the safety and effectiveness of the device. The FDA is actively examining how regulatory decision-making can keep up with rapid device and data science developments.

### Patient Acceptance and Involvement

A reasonable assumption is that patients and family members will become increasingly comfortable with technology. However, that does not lessen the importance of making the technology nonobtrusive and simple to use. The patient may find that access to the data provided by the device is empowering; reports of their own data can be provided to the patient, and alerts could be provided for concerning events, such as a seizure. This engages the patient as a partner in treatment and provides a new means of communication between patient and physician.

### Ethical Concerns

Neurotechnology interfaces have the potential to move beyond treatment of disease to the enhancement of natural human abilities by improving the flow of information between minds, bodies, machines, connected computers, and the physical world. This gives rise to social, legal, and ethical concerns.

Fundamental ethical principles, including those of identity, autonomy, and agency, must be considered. If actions are mediated by “smart” neurotechnology, can they be said to be autonomously intended? If behavior is artificially affected by neurotechnology, can the person be held accountable for the consequences that arise from that behavior? If neurotechnology interventions are predicated on only one understanding of “normal,” does it jeopardize the person's freedom to choose differently and so abuse that person’s human rights? If neurotechnology has the potential to change thoughts, emotions, and behaviors, who should govern its development and access? Is equality of access compromised when an individual with means acquires a neurotechnology to artificially enhance abilities? Does this introduce unfairness and risk widening social inequality?

## Implications for the Neurologist and Planning for the Future

The neurologist in 2035 will have access to a number of safe and effective neuromodulation devices that provide specific, targeted, and modifiable treatment for the majority of neurologic and psychiatric disorders. Noninvasive testing, such as EEG, fMRI, diffusion tensor imaging, and TMS, and less invasive methods of direct brain recording, such as sEEG, will help to identify circuit pathology and thus target the best location for neuromodulation intervention. The treatment plan will consider whether patients are most likely to benefit from pharmacotherapy, resective or ablative procedures, noninvasive or invasive neuromodulation, or some combination of each. Other therapies will be synergistically combined, such as physical therapy and cerebellar stimulation after stroke, amygdala stimulation with desensitization in PTSD, and, perhaps, fornix or entorhinal stimulation with monoclonal antibodies for Alzheimer disease.

Many neuromodulation devices will be used as disease management platforms. Patients and physicians will be empowered by device-provided objective neural biomarker data to personalize device programming and track the clinical response, not only to neuromodulation, but also to changes in behavior or in pharmacologic treatment. Data will be incorporated from peripheral devices that monitor heart rate, respiratory rate, sleep, and activity, and include patient data entry capabilities. Dashboards will show physicians trends in electrophysiologic biomarkers that are early indicators of disease activity, before patients experience symptoms. These biomarker trend data will be used to predict whether pharmacologic, surgical, or device-specific changes in therapy will be of benefit, and how the therapeutic measures might be optimized in a patient-specific manner based on large databases of prior patient and clinical experience. Alerts will be provided to the physician and to the patient when there are concerning changes in brain or peripheral device data and patients may be instructed to take a specific action, such as to take a medication.

Current neuromodulation treatments have proven benefit, a number will demonstrate benefit soon, and others will follow. The neurologist today is privileged to be in the midst of a time of rapid development of personalized and flexible technologies that deliver data and provide powerful therapeutic options for patients with neurologic disorders.

## Glossary

DBS: deep brain stimulation
FDA: Food and Drug Administration
OCD: obsessive-compulsive disorder
PTSD: posttraumatic stress disorder
tACS: transcranial alternating current stimulation
tDCS: transcranial direct current stimulation
TMS: transcranial magnetic stimulation
